# Association of Guillain–Barré Syndrome With Nonavalent Human Papillomavirus Vaccine: A Postmarketing Drug Safety Study

**DOI:** 10.1155/ogi/1853905

**Published:** 2026-05-31

**Authors:** Mario Arturo González Mariño

**Affiliations:** ^1^ Department of Obstetrics and Gynecology, Faculty of Medicine, Universidad Nacional de Colombia, Bogotá, Colombia, unal.edu.co

**Keywords:** adverse drug event, guillain–barré syndrome, human papilloma virus vaccine, uterine cervical neoplasms

## Abstract

**Introduction:**

Human papillomavirus (HPV) vaccines prevent types of HPV responsible for most cervical cancers. While Guillain–Barré syndrome (GBS) has rarely reported postvaccination, a causal relationship remains unestablished. This study evaluates the association between the nonavalent HPV vaccine (9‐valent) and GBS using the Vaccine Adverse Event Reporting System (VAERS).

**Methods:**

We analyzed VAERS data from January 1, 2016, to July 26, 2024, focusing on domestic reports for individuals aged 6–29 years. GBS cases were identified using the MedDRA preferred term code 10018767. Disproportionality was assessed via a 2 × 2 contingency table to calculate the proportional reporting ratio (PRR) and Yates’ chi‐squared (*χ* ^2^) test. A signal was defined as PRR ≥ 2, *χ* ^2^ ≥ 4, and *n* ≥ 3.

**Results:**

A total of 11 serious GBS cases were reported following HPV 9‐valent vaccination out of 14,367 total adverse events for the vaccine. Comparison with all other vaccines (1757 GBS cases) yielded a PRR of 0.79 (95% CI: 0.44–1.44) and a Yates’ *χ* ^2^ of 0.384 (*p* = 0.535). Of the HPV 9‐valent–associated GBS cases, 64% occurred in females and 57% in the 6–17 age group. Most cases occurred 10–30 days postvaccination; no deaths were reported.

**Conclusions:**

The findings indicate an absence of a disproportionality signal in VAERS, suggesting that GBS is not reported more frequently after HPV 9‐valent than other vaccines. However, this study is limited by a small number of cases and the inherent constraints of passive surveillance; thus, these results reflect reporting patterns and cannot establish causality or absolute incidence.

## 1. Introduction

Infection by human papillomavirus (HPV) is associated with benign and malignant diseases. Among them, cervical cancer is particularly relevant because of its high impact on public health. Each year in the United States, approximately 13,000 new cases of cervical cancer are diagnosed, and approximately 4000 women die of this cancer [1].

The HPV vaccine protects against the types of HPV that most often cause cervical cancer. It is expected that with the nonavalent vaccine (9‐valent), the prevention of cervical cancer will increase from 66.2% to up to 90% [2]. The CDC recommends that the 9‐valent vaccine be administered to preteens aged 11–12 years, but it can be given starting at age 9 through 26 if they are not already vaccinated [3]. It corresponds to second‐generation HPV vaccines after bivalent and quadrivalent. As of late 2016, the HPV 9‐valent has been the only available vaccine in the United States [4].

More injection‐site adverse events have been reported in the 9‐valent vaccine than in the HPV quadrivalent group [5]. The 9‐valent vaccine contains a greater amount of HPV virus‐like particle antigens and aluminum hydroxyphosphate sulfate, which are used to potentiate the immune response to an antigen [6]. However, both HPV quadrivalent and 9‐valent vaccines had similar frequencies of systemic adverse events, such as headache, pyrexia, fatigue, and nausea: 55.8% of women who received the 9‐valent vaccine and 54.9% of women who received quadrivalent vaccine [6].

A postmarketing surveillance study using spontaneous reports found that Guillain–Barré syndrome (GBS) was reported more frequently following the administration of quadrivalent HPV vaccine. In 70% of the patients whose vaccination dates were known, symptoms began within 6 weeks after vaccination [7]. However, a more recent review concludes that there was evidence that suggests HPV vaccination was not associated with an increased risk of GBS [8].

GBS comprises a group of peripheral nerve disorders characterized by weakness or paralysis, which is believed to have an autoimmune etiology. It is the most common cause of acute flaccid paralysis worldwide [9]. GBS was associated with an adverse event following immunization (AEFI) in 1976 with influenza vaccine (A/New Jersey; “swine flu”) [10].

A vaccine is a drug. Like any drug, vaccines have benefits and risks. However, serious vaccine reactions are extremely rare [11]. The Vaccine Adverse Event Reporting System (VAERS) is a nationwide passive reporting system with an epidemiological database that collects information about adverse events that occur after the administration of licensed vaccines in the United States [12].

The proportional reporting rate (PRR) is a method used to measure the proportion of notifications in a database that contains a particular combination of a drug and an event. In the same database, this ratio is compared with notifications of the same event but with the other drugs. If the PRR for a particular drug‐event combination is significantly high and is not a known reaction, it may represent a sign [13].

## 2. Methods

### 2.1. Data Source

Data were extracted from VAERS using the CDC WONDER (Wide‐ranging Online Data for Epidemiologic Research) interface. To identify cases of GBS, the Medical Dictionary for Regulatory Activities (MedDRA) standardized coding system was utilized. Specifically, the search was restricted to the MedDRA preferred term code 10018767 (GBS). The dataset was formally reviewed and finalized for analysis on August 10, 2024.

### 2.2. Inclusion and Exclusion Criteria

The study population included reports of serious adverse events following the administration of the 9‐valent HPV vaccine and of all other vaccines specially those reported as GBS, between January 1, 2016, and July 26, 2024. Patients were included based on standard clinical recommendations and CDC WONDER predefined groupings: 6–17 years (primary adolescent target) and 18–29 years (young adult catch‐up population).

Exclusion Criteria: Nondomestic (foreign) reports were manually excluded to ensure data consistency and follow‐up reliability within the U.S. healthcare system.

### 2.3. Data Cleaning and Integrity

Data cleaning was performed within the CDC WONDER system by filtering for unique VAERS ID numbers. This ensured that the unit of analysis was the individual clinical event rather than the total count of reported symptoms, preventing the overcounting of cases. VAERS reports are subject to CDC/FDA internal quality control procedures prior to public release. For this analysis, duplicate entries were minimized by filtering for unique VAERS identification numbers within CDC WONDER.

### 2.4. Handling of Coadministration

While this study focused on the 9‐valent HPV vaccine as a suspect exposure, reports involving multiple vaccine exposures (concurrent vaccinations) were not excluded. This approach was selected to provide a comprehensive, descriptive overview of GBS reports, acknowledging the common practice of coadministering vaccines in the target age groups.

### 2.5. Statistical Analysis

A 2 × 2 contingency table was constructed to evaluate the reporting frequency. The PRR and Yates’ Chi‐squared test were calculated to identify potential safety signals. A PRR ≥ 2, a Chi‐squared value ≥ 4, and a case count ≥ 3 were used as the threshold criteria for a significant signal.

The 2 × 2 contingency table was constructed comparing the following:

 Reports of GBS following 9‐valent HPV vaccination.
 Reports of GBS following all other vaccines.
 Non‐GBS adverse event reports in each group.

Disproportionality analysis was assessed using the PRR and two‐sided Yates‐corrected Chi‐square test. A *p* value < 0.05 was considered statistically significant.

The PRR was calculated as

(1) PRR=a/a+cb/b+d,

where *a* = number of GBS reports following 9‐valent HPV vaccination. *b* = number of GBS reports following all other vaccines. *c* = number of non‐GBS adverse event reports following 9‐valent HPV vaccination. *d* = number of non‐GBS adverse event reports following all other vaccines.

The 95% confidence interval (CI) for the PRR was calculated using standard logarithmic transformation methods. Yates’ continuity correction was applied to the Chi‐square statistic due to small cell counts. PRR analysis identifies disproportional reporting patterns but does not establish causality.

Statistical calculations, including 95% CIs, were performed using Microsoft Excel (Microsoft Corp., Redmond, WA). The PRR and Chi‐square calculations with Yates’ correction were verified using the Calculado.net large number computational tool.

### 2.6. Ethical Considerations

• No Institutional Review Board approval is required because data are deidentified and publicly available
• No patient‐level data were accessed
• Study conforms to principles of postmarketing surveillance research

## 3. Results

A total of 14 reports of GBS were initially identified during the clinical review of the VAERS database. However, for the purpose of the formal disproportionality analysis (PRR and *χ* ^2^), the sample was restricted to 11 serious cases in accordance with the study’s inclusion criteria focusing on serious adverse events. The 3 excluded reports were classified as nonserious by VAERS.

Based on the data presented in Table 1, 11 cases of GBS were reported following 9‐valent HPV vaccination. During the same period, 14,356 other AEFIs were reported for the 9‐valent HPV vaccine, and 1757 GBS cases were reported following all other vaccines. The calculated PRR was 0.79 (95% CI: 0.44–1.44), and the Yates’ *χ* ^2^ was 0.384 (*p* = 0.535). Because the PRR was less than 2.0 and the *χ* ^2^ was less than 4.0, these findings do not meet the threshold for a disproportionality signal. Among the initial 14 reported GBS cases with available clinical details (Table 2), eight occurred in individuals aged 6–17 years and six in those aged 18–29 years. Nine cases occurred in females and five in males. Eleven cases were classified as serious (nonfatal), three as nonserious, and no deaths were reported. Regarding time‐to‐onset, data were extracted directly from VAERS reports. The highest frequency of onset occurred between Days 10–14 and 15–30 (3 cases each). No deaths were reported.

**TABLE 1:** Serious adverse events reported in the VAERS database (January 1, 2016 to July 26, 2024) among individuals aged 6–29 years.

	9‐valent HPV vaccine	All other vaccines	Total
Guillain–Barré S.	11^a^	1757^b^	1768
Other AEFIs	14,356^c^	1,825,303^d^	1,839,659
Total	14,367	1,827,060	1,841,427

Abbreviations: AEFI = adverse event following immunization; VAERS = vaccine adverse event reporting system.

^a^Number of GBS reports following 9‐valent HPV vaccination.

^b^Number of GBS reports following all other vaccines.

^c^Number of non‐GBS adverse event reports following 9‐valent HPV vaccination.

^d^Number of non‐GBS adverse event reports following all other vaccines.

**TABLE 2:** Characteristics of all reported cases of Guillain–Barré syndrome by age group (N = 14), January 1, 2016, to July 26, 2024.

Characteristics	6–17 years (*n* = 8)	18–29 years (*n* = 6)	Total *N* = 14^∗^
*Sex*			
Male	2	3	5
Female	6	3	9

Seriousness*^∗∗^*			
Nonserious	3	0	3
Serious, nondeath	5	6	11
Serious, death	0	0	0

Time to onset (days)*^∗∗∗^*			
0–2	0	2	2
3–5	2	0	2
6–9	0	0	0
10–14	2	1	3
15–30	2	1	3
31–60	0	1	1
61–120	0	0	0
> 120	1	1	2
Unknown	1	0	1

^∗^Total includes 11 serious adverse events and 3 nonserious adverse events reported to VAERS.

^∗∗^Seriousness classification as reported in VAERS.

^∗∗∗^Time to onset was calculated as the interval (in days) between vaccination date and symptom onset date as recorded in VAERS. When either date was missing or incomplete, time‐to‐onset was classified as “unknown.” The intervals were categorized based on standard pharmacovigilance reporting windows provided by VAERS.

According to the established signal detection criteria (PRR ≥ 2, number of cases ≥ 3, and *χ* ^2^ ≥ 4), these findings do not meet the threshold for a disproportionality signal. The PRR below 1.0 indicates that GBS was not reported more frequently following 9‐valent HPV vaccination compared with other vaccines in the database during the study period. However, this result should be interpreted as the absence of a statistical signal rather than evidence of the absence of risk.

## 4. Discussion

The safety of the 9‐valent HPV vaccine is essential for maintaining public confidence in cervical cancer prevention programs. Pharmacovigilance through systems such as VAERS is vital for identifying rare or previously unrecognized adverse events [14, 15].

Data mining methods have been proposed as screening tools for improving the efficiency of adverse event reporting. The PRR is a statistic that is used to generate a signal about the potential hazard of drugs [16]. In this analysis, the reporting pattern of GBS following HPV 9 valent vaccination from 2016 to 2024 did not suggest a safety concern. Interpretation of statistical signal using a standardized signal detection threshold (PRR ≥ 2, number of cases ≥ 3, and *χ* ^2^ ≥ 4) helps prevent “false alarms” caused by isolated reports [16]. The results, specifically a PRR of 0.79 and a two‐sided Yates’ Chi‐squared value of 0.384, fall well below these criteria. Because the PRR behaves similarly to a relative risk, a value below 1.0 indicates that GBS is not reported more frequently for HPV 9‐valent than for the background of other vaccines in the 6–29 age group [17]. Therefore, while GBS has been historically associated with various immunizations, our findings suggest that HPV 9‐valent maintains a favorable safety profile regarding this specific neurological condition.

While these results provide valuable insights, they must be interpreted within the inherent constraints of the VAERS database. As a passive surveillance tool, VAERS is susceptible to reporting biases and underreporting, particularly for nonserious events, though serious outcomes such as GBS tend to be reported more consistently [18, 19]. A notable limitation of this study is the small number of cases identified (*n* = 11), which introduces a degree of statistical uncertainty and may limit the power to detect rare signals. Furthermore, because analyses lack denominator data regarding the total doses administered, these findings reflect reporting patterns rather than true incidence rates, and absolute risk cannot be calculated. The analysis is also limited by potential misclassification and an inability to establish direct causality. Additionally, the potential for confounding is expected because coadministration with other vaccines was not excluded; therefore, adverse events cannot be attributed uniquely or exclusively to the 9‐valent HPV vaccine. Finally, while CDC/FDA cleaning procedures were utilized, residual duplicate reporting and the use of predefined age stratifications may further impact comparability with other studies.

## 5. Conclusions

The analysis found no statistically significant differences in the reporting of GBS following the 9‐valent HPV vaccine compared to other vaccines. While these findings indicate an absence of a disproportionality signal in VAERS, it is important to note that VAERS data cannot be used to establish causality or determine the incidence of an adverse event. Therefore, controlled epidemiologic studies are needed to confirm these findings.

## Funding

The author has nothing to report.

## Ethics Statement

The author affirms that this manuscript is an honest, accurate, and transparent account of the study being reported; that no important aspects of the study have been omitted; and that any discrepancies from the study as planned (and, if relevant, registered) have been explained.

## Conflicts of Interest

The author declares no conflicts of interest.

## Data Availability

Data were sourced from CDC WONDER (https://wonder.cdc.gov/vaers.html). Because the VAERS database is updated monthly, the results presented here are specific to the data available during the final review on August 10, 2024.
